# Maximizing Opportunities: Family Planning and Maternal, Infant, and Young Child Nutrition Integration in Bondo Sub-County, Kenya

**DOI:** 10.1007/s10995-017-2341-9

**Published:** 2017-08-01

**Authors:** Chelsea M. Cooper, Angella Ogutu, Everlyn Matiri, Hannah Tappis, Devon Mackenzie, Anne Pfitzer, Rae Galloway

**Affiliations:** 1 Maternal and Child Survival Program, Washington DC, USA; 2 Jhpiego, Baltimore, USA; 3 Jhpiego, Nairobi, Kenya; 4 Maternal and Child Survival Program, Nairobi, Kenya; 5 PATH, Nairobi, Kenya; 6 PATH, Washington DC, USA

**Keywords:** Integrated service delivery, Family planning, Maternal nutrition, Infant nutrition, Kenya, Breastfeeding, Lactational amenorrhea method

## Abstract

*Purpose* This article shares learning from an innovative demonstration program integrating maternal, infant, and young child nutrition (MIYCN) and family planning (FP) services in western Kenya, providing recommendations for future work to expand MIYCN and FP integration. *Description* Six health facilities reorganized to integrate MIYCN and FP services and community health volunteers (CHVs) promoted MIYCN and FP in adjacent communities in Bondo Sub-County over a 1-year period. At the facility level, each provider was directed to provide both sets of services in a single room during FP, antenatal care, postnatal care, or child consultation visits (a “one stop shop” approach). At community level, CHVs were to conduct household visits equipped with new integrated materials and incorporate MIYCN and FP within community activities. *Assessment* Although the “one stop shop” approach, where one provider offers all integrated services in one room, was initially proposed for all facilities, this worked most effectively in the dispensary and health centers. The sub-county hospital adapted the approach such that integrated services were offered by more than one provider during a visit, with clients linked from one provider to another through same-day intra-facility referrals. CHVs were generally able to incorporate MIYCN and FP content within household visits and community activities; however some knowledge gaps were noted after initial training, necessitating additional refresher training. *Conclusion* This demonstration experience revealed that future replication efforts should enable sub-county team leadership, assess facility readiness, streamline data collection, build local buy-in, and prioritize dispensaries and health centers with high client loads.

## Significance

This paper describes the learning from a demonstration initiative implemented in Bondo Sub-County, Kenya which integrated family planning and maternal, infant, and young child nutrition information and services. The findings of this paper can be used to inform future integration efforts in Kenya and similar settings.

## Purpose

Family planning (FP) and maternal, infant, and young child nutrition (MIYCN) are critical components of care for women during pregnancy, childbirth, and their child’s first 2 years of life. Optimal FP and MIYCN practices have mutually beneficial effects on maternal and child health and nutrition. Meta-analyses demonstrate that close birth intervals increase the risk of neonatal, child, and maternal mortality; stunting in children; and poor pregnancy outcomes (Conde-Agudelo et al. 2006; Rutstein 2005). Additionally, exclusive breastfeeding (EBF) in the first 6 months not only protects the infant from illness and malnutrition, but also meets the mother’s contraceptive needs if she practices the lactational amenorrhea method (LAM)^1^. Optimal breastfeeding of infants under age two has the greatest potential impact on child survival of all preventive interventions, with the potential to prevent 13% of all deaths in children under five in the developing world (Jones et al. 2003). LAM is over 98% effective as a contraceptive method when used correctly (Labbok et al. 1997), and programs that have promoted LAM as part of postpartum family planning (PPFP) counseling along with infant and young child feeding messages have demonstrated increases in EBF duration and contraceptive use at 1 year postpartum (Ahmed et al. 2015; Bongiovanni et al. 2005). Offering infants foods or liquids other than breastmilk leads to reduced frequency and duration of breastfeeding, which can trigger ovulation, and results in LAM no longer being a viable FP option. Thus, initiating use of another modern contraceptive method before introducing complementary food or liquids at 6 months is especially critical for preventing closely spaced pregnancies. Harmonizing MIYCN and FP counseling and services throughout the continuum from pre-pregnancy to early childhood can reduce missed opportunities for provision of comprehensive services for woman and child.

In Kenya in 2009^2^, half of all second or higher order pregnancies occurred within intervals of less than 24 months after the preceding birth (Moore et al. 2015). Only 32% of children under 6 months of age were exclusively breastfed, and median duration of EBF was estimated at less than 1 month (Kenya National Bureau of Statistics and ICF Macro 2010). Among women within 2 years postpartum, 58% had unmet need for FP (Maternal and Child Health Integrated Program 2012). At the time the intervention was designed, Nyanza Region had lower contraceptive prevalence, higher total fertility, lower median duration of exclusive breastfeeding, and higher rates of infant and under-5 mortality than Kenyan national rates (Kenya National Bureau of Statistics and ICF Macro 2010). In 2008/2009 Nyanza had the highest percentage of married women with an unmet need for FP, at 32%.

At the policy level, the Kenyan Ministry of Health (MOH) has mandated integrated services since 2009, with the National Reproductive Health and HIV&AIDS Integration Strategy (Kenya National AIDS Control Programme and Division of Reproductive Health 2009), however detailed operational guidelines did not address clearly the FP and nutrition elements. National MOH stakeholders welcomed the prospect of learning about integrated FP and nutrition service delivery.

The Kenyan health system supports delivery of maternal and child health and other services through dispensaries, health centers, and hospitals. FP and nutrition services are often provided in parallel in different rooms and/or by different providers. As a complement to the facility-based services, community health volunteers (CHVs) provide health education, services, and facility referrals as needed to households in their catchment areas. At the time of the intervention, CHVs provided contraceptive pills and condoms, and referred clients to health facilities for other FP methods. CHVs are supervised by a community health extension worker (CHEW) who serves as the link between CHVs and the health facility. Each CHEW supervises approximately 25 CHVs. While CHVs are expected to deliver an integrated package of information and services, gaps in counseling on FP and nutrition exist and synergies between nutrition and FP are not adequately emphasized.

As part of an effort to address unmet need for FP and improve MIYCN practices, the USAID-funded Maternal and Child Health Integrated Program (MCHIP), in collaboration with the Kenya Ministry of Health, introduced an innovative demonstration program to integrate MIYCN and FP counseling and services in selected sites in Bondo Sub-County in 2011. The primary objective of the intervention was to take advantage of the synchronic timing of PPFP and MIYCN messages and interventions to enhance and strengthen linkages between these services.

The purpose of this paper is to establish the feasibility of integrating FP and MIYCN into existing antenatal, maternity, postnatal, and child health services. Recommendations are offered for future work to expand MIYCN and FP integration in Kenya and similar settings.

## Description

### Program Description

The demonstration took place in six health facilities and adjacent community units in Bondo Sub-County, Siaya County, Nyanza Region over the course of 1 year (Table 1). Intervention sites included: Bondo Sub-County Hospital (catchment: 7040 households)^3^, Ogam Dispensary (catchment: 1029 households), and Kapiyo (catchment: 1058 households), Usigu (catchment: 1755 households), Got Matar (catchment: 1795 households), and Gobei (catchment: 1385 households) health centers. Guided by a formative assessment involving interviews with facility service providers and CHVs and focus group discussions to gather community perspectives, the program adopted a multi-level approach addressing facility, community and household levels. The approach reinforced and increased exposure to MIYCN and FP services, as well as information about the importance of EBF during the first 6 months after birth, timely postpartum contraceptive uptake, PPFP options including LAM, continuation of breastfeeding after introducing complementary foods at 6 months, and transition to another modern FP method before LAM criteria are no longer met.

**Table 1:** Time frame and key activities for the MIYCN-FP integration initiative in Kenya

Activity	Timeline
Initial advocacy with Ministry of Health teams	Early 2011
Formative assessment	June–July 2011
Planning and design meeting	August 2011
Pre-testing and finalization of social and behavior change communication materials	September–October 2011
Training of service providers and community health volunteers at Bondo Sub-County Hospital	July 2012
Implementation begins at Bondo Sub-County Hospital	August 2012
Training of service providers and community health volunteers at additional sites	February 2013
Implementation begins at additional sites	February 2013
Supportive supervision visits	September 2012–February 2014
PPFP integration study conducted	June 2014

The program employed a “one stop shop” approach at selected facilities, where clients received critical MIYCN and FP information and services during antenatal, intrapartum, child welfare clinic (CWC), and FP consultations. The intended approach involved: (1) realigning services so that clients could receive MIYCN and FP services in the same room, offered by the same provider (without referral); (2) training service providers and providing ongoing supportive supervision on MIYCN and FP in the six health facilities; and (3) introducing evidence-based MIYCN-FP communication materials (including a job aid, poster, and brochure) to support provision of integrated services. Due to limited funding, birth attendants were not trained in nor could offer immediate postpartum IUDs and tubal ligation. However, in order to respond to observed gaps in provision of long-acting FP methods, providers were offered coaching and continuing medical education on these skills during the program period.

At community level, the program sought to strengthen existing community FP and nutrition activities. The approach involved: (1) training CHVs and their supervisors, CHEWs, on the links between MIYCN and FP and how to integrate the two services; (2) engaging CHEWs and Sub-County Health Management Teams (SCHMTs) to provide supportive supervision to CHVs; (3) orienting CHVs and CHEWs on use of existing nutrition and FP materials and newly developed MIYCN-FP communication materials (counseling cards, brochure, and poster); and (4) incorporating MIYCN-FP within existing community activities, such as CHV home visits, community dialogues, mother support groups, and health action days.

Training for facility providers and CHVs used participatory approaches with training modules that included content on the rationale for integration (including benefits of integration for health workers), key MIYCN and PPFP concepts, common barriers and facilitators for use of nutrition and FP services, an overview of strategies for integrating services, orientation to the communication materials and supplemental registers, practical review, and action planning.

### Learning Approaches

Findings and lessons learned are drawn from multiple sources (Table 2). After implementation began, MCHIP and the SCHMT conducted quarterly supportive supervision visits at implementation sites. A standardized MIYCN-FP supportive supervision tool was administered during regular supervision visits at facility and community levels. While MCHIP developed MIYCN-FP supplemental registers for facility providers and CHVs, inconsistencies in use of registers across sites resulted in insufficient data making it difficult to use this data to analyze program performance. MCHIP conducted a multi-site study on factors facilitating and hindering implementation and scale-up of PPFP integration in Kenya and India, including sites in Bondo Sub-County where the MIYCN-FP initiative took place. The study used a cross-sectional design to track services for pregnant women and women with children up to 2 years old received during the course of their visit to a health facility to determine whether PPFP counseling and/or services were offered in an integrated manner. This methodology, called client flow assessment, was developed and tested by the Integra Initiative (Birdthistle et al. 2014). The study also included structured exit interviews (n = 71) with clients seeking ANC, PNC, child welfare, FP services, and HIV counseling and care, as well as a qualitative component including in-depth interviews with sub-county representatives (n = 4), facility in-charges (n = 6), and service providers (n = 4). Research protocols for this study were reviewed by the Johns Hopkins Institutional Review Board.

**Table 2:** **Data Sources**

Data source	Description
**Routine data collection sources**	
MIYCN-FP facility supplemental registers	Supplemental registers collect data on MIYCN-FP practices and counseling/services/referrals offered; completed during each contact by MCH and FP providers
MIYCN-FP community supplemental registers	Supplemental registers collect data on MIYCN-FP practices and counseling/services/referrals offered; completed during each household visit by CHVs
Supportive supervision reports	Prepared quarterly by MCHIP program staff based on findings from application of standardized MIYCN-FP supervision tool
Health information system	Service statistics on breastfeeding and contraceptive use at focus sites obtained from Kenya health information system
**PPFP integration study sources**	
Client flow form	Research assistants screened all female clients arriving at target health facilities over a period of one week. Clients who were pregnant and/or had a child less than 2 years of age were asked to carry a checklist throughout their visit at the health facility, on which providers and staff marked the services and referrals provided to the client. The research team documented the time the client arrived at the service area on the form and then gave the form to the client and asked her to give it to any facility staff she encountered, regardless of whether they were clinicians or worked in ancillary services.
Client exit interviews	Structured exit interviews conducted with clients seeking ANC, PNC, child welfare, FP services, and HIV counseling and care.
Facility in-charge in-depth interviews	Structured interviews conducted with facility in-charges at intervention facilities
Service provider in-depth interviews	Structured interviews conducted with service providers at intervention facilities
Community-based provider in-depth interviews	Structured interviews conducted with community health volunteers working in the intervention community units
Sub-County representative informant interviews	Structured interviews conducted with Sub-County Health Management Team and other sub-county stakeholders

## Assessment

### Implementation Variations Across Sites

**Fig. 1:**
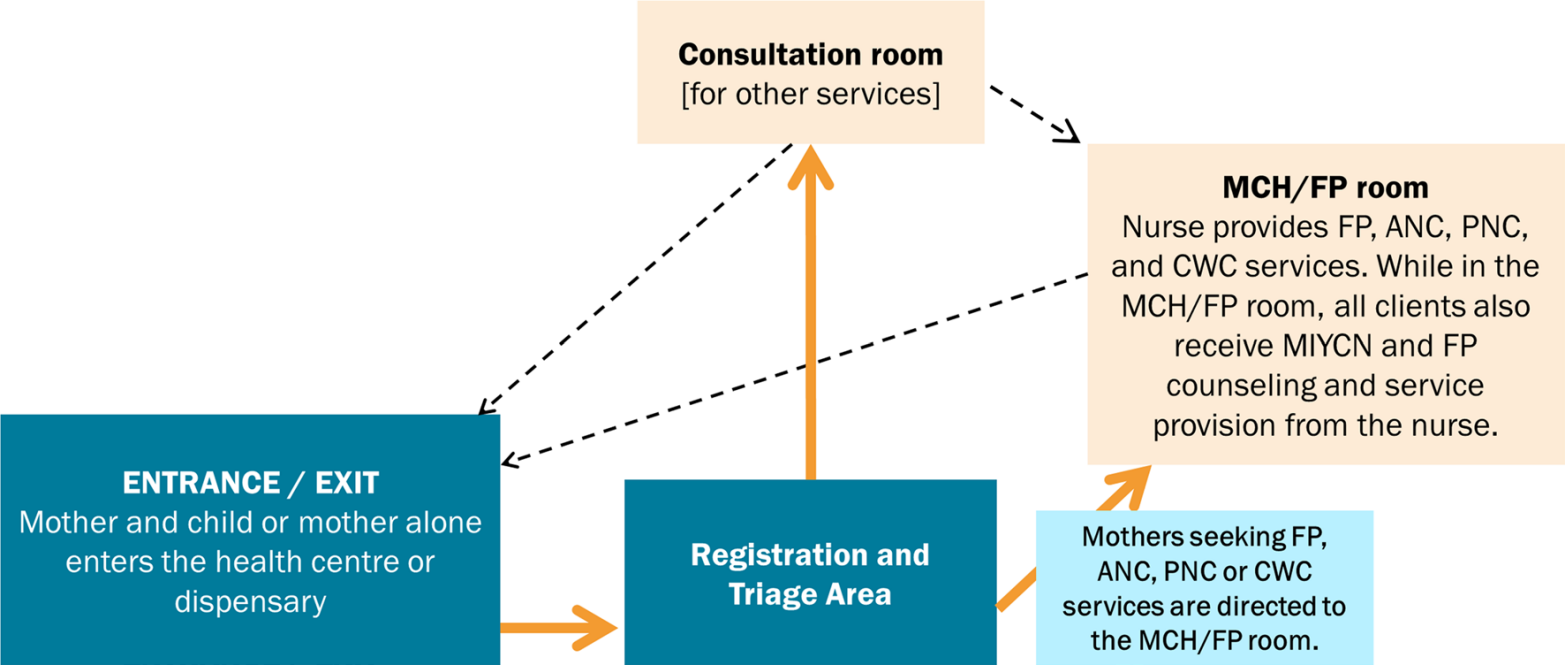
Client flow at the health center/dispensary level (*PMTCT services are integrated within maternal and child health service provision*)

**Fig. 2:**
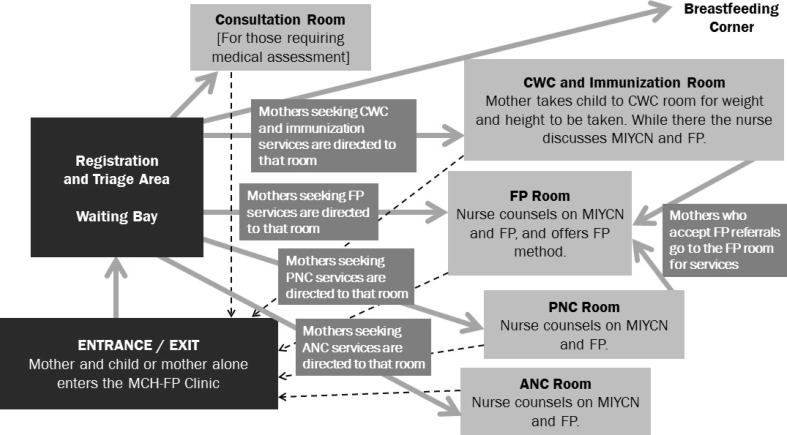
Client flow at the sub-county hospital (*PMTCT services are also integrated within maternal and child health service provision*)

The *“*one stop shop*”* approach (Fig. 1), initially proposed for all of the health facilities, worked most effectively at the dispensary and health centers. Bondo Sub-County Hospital adopted an approach whereby integrated services were generally offered by more than one service provider within the facility during the same visit, and clients were linked from one provider to another through same-day intra-facility referrals, or sometimes the same provider moved with the client to another room to offer the service there (Fig. 2). The modification of the approach at the sub-county hospital was deemed necessary due to the lack of human resource continuity, infrastructure, and workloads at that site. In Bondo Sub-County Hospital, unlike at the dispensary and health centers, service providers rotate annually. As a result, service providers working in MCH/FP and the maternity and outpatient departments who had been trained in MIYCN-FP integration were moved to other service delivery departments. Consequently, MCHIP had to train additional staff and provide onsite mentorship for staff in MCH/FP in the maternity and outpatient departments to ensure continuity in MIYCN-FP services. At the dispensary and health centers, staff continuity posed less of a challenge and providers trained on MIYCN-FP integration were more likely to orient colleagues on the integration process and use of materials than at the sub-county hospital.

### Findings Following Implementation

Table 3 presents findings from the PPFP Integration Study’s client flow assessment. As the table shows, the lower level health facilities (health centers) demonstrated greater integration of nutrition and FP counseling and services during client visits than at the hospital. At Bondo Sub-County Hospital, out of all visits, 9.2% of pregnant women and those with a child less than 2 years old received *both* nutrition and FP services during their visit, whereas at the two health centers, 44.7% of women received both services. Across visits where clients accessed antenatal care, postnatal care, and child health services, there was substantially more counseling on MIYCN, FP, and LAM conducted at health centers than at the hospital. Supportive supervision also revealed that service providers often gave more emphasis to the primary service that brought a client to the facility than any secondary service.

**Table 3:**
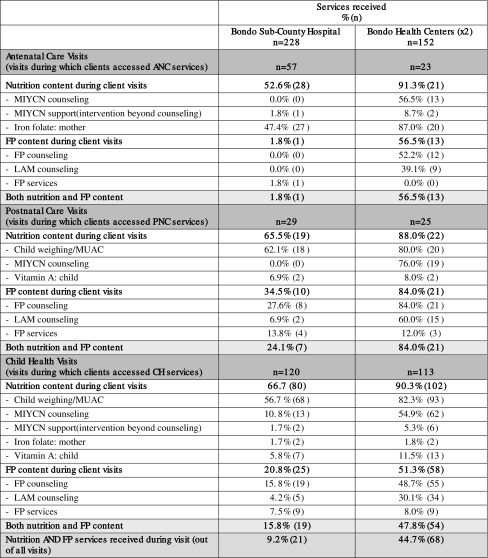
Nutrition and family planning services received by service delivery area accessed during visit, as determined through client flow assessment

Findings from client exit interviews indicate a positive response among clients to being able to access family planning while at the health facility for other services. Seventy-nine percent of clients interviewed who were offered FP services during their visit (for ANC, labor, PNC, and child welfare) thought it improved quality of services. No respondents perceived that it decreased the quality of services. When asked how knowing that FP services were available affected their willingness to come to the clinic that day, 74% of clients said that it increased their willingness; no clients indicated that it decreased their willingness to come for services that day.

During supportive supervision visits, most service providers demonstrated knowledge of MIYCN-FP integration. However, supervision visits after the initial training revealed gaps in CHV knowledge about PPFP, especially in regard to return to fecundity, LAM criteria, and the transition to other modern methods. Supervisors noted that health workers gave more emphasis to breastfeeding as compared to complementary feeding and maternal nutrition information. As a result, refresher trainings were held across sites to discuss challenges and address knowledge gaps. After refresher trainings, supervision teams noted enhanced CHV counseling skills and ability to convey MIYCN-FP information.

Supervision visits revealed that except for IUDs and female condoms, FP commodities were generally available at the six health facilities. Temporary stock-outs of implants occurred at some facilities in light of widespread demand for this method. One facility in-charge noted, “There is still need to train providers in the maternal department on how to administer long-term FP methods, which all of them lack [training on].”

Health facility staff generally recognized benefits of integrating services. For example, one health center in-charge noted “… for the women who come this side, it is an advantage to them because it is time saving… because rather than telling them to go to the FP room after coming here, she gets everything in one room, one stop shop.” Another provider mentioned, “The client is able to get this method without necessarily moving from one point to another point…it has also reduced the waiting time of this client.” However, several staff and facility in-charges reported concerns with workload associated with the integrated approach. Infrastructure limitations, including lack of availability of dedicated private space for provision of integrated services, were also cited as a barrier to the “one stop shop” approach.

Facility providers and CHVs experienced challenges with completing the supplemental registers. During an early round of supportive supervision, these registers were observed to be only partially completed, not complete, or could not be traced. Health workers indicated that the supplemental ledger added to their workload and they prioritized completing either the government registers or the supplemental register. Over time and with coaching during the supervision visits, improvements in data collection were noted, but not to a level where data from those registers could be used to monitor program performance.

### Behavior Change and Service Use

**Fig. 3:**
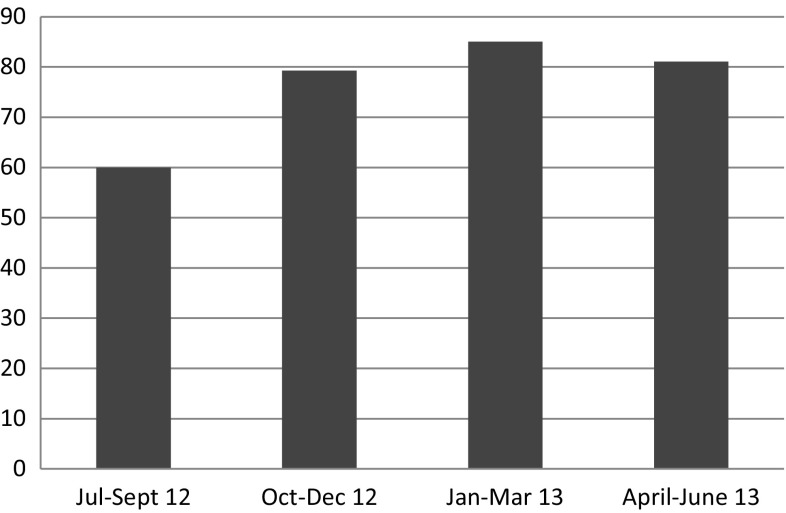
Percent of children under 6 months exclusively breastfed, Bondo Hospital. Source:Kenya Community Health Information System

During supportive supervision, providers reported marked increases in the number of FP clients at demonstration sites since the rollout of integrated services. The Bondo Contraceptive Prevalence Rate (CPR) Survey, a representative survey of the whole sub-county conducted in 2014, revealed that Bondo had an impressively high CPR, considerably higher than the Nyanza Province (now Nyanza Region) rates from the KDHS 2008–2009. LAM represented 4% of the contraceptive method mix, whereas the KDHS 2008–2009 revealed less than 1% use of LAM within the method mix in Nyanza (Jhpiego Kenya Country Office for MCHIP Kenya 2014; Kenya National Bureau of Statistics and ICF Macro 2010). Community health information system data also reveal an increase in exclusive breastfeeding for Bondo Sub-County Hospital over the course of July–September 2012 through April–June 2013 (Fig. 3) (Ministry of Health 2014). This period correlates with the course of implementation of the integrated approach. While it is not possible to directly link integration efforts with these positive outcomes, changes are likely attributable to the broader, multi-pronged and collaborative set of activities implemented in the sub-county, which included the MIYCN-FP integration approach in selected sites.

In the PPFP Integration Study, when asked about the most significant changes that had occurred in Bondo since the service integration, key informants mentioned increased contraceptive use, reduction of giving food and liquids other than breast milk before 6 months of age, increases in EBF, increased client and provider knowledge about nutrition and FP, increased number of CHV follow-up visits with clients, improvements in health-seeking behavior, improved health worker documentation and recordkeeping, enhanced coordination and collaboration across stakeholder groups, and reduced wait times for clients. One sub-county official noted, “Communities are now well enlightened on FP because of the good sensitization by CHVs.” One CHV mentioned, “FP-MIYCN improves the well-being of the mother and child. Some mothers reported their children did not get frequent infections when they exclusively breastfed their children.” Other CHVs also reported that women who chose to use LAM expressed a willingness to transition to other FP methods when infants turned 6 months old. It should be noted that one woman mentioned that service providers required proof of menses before offering an FP method. She explained, “A few service providers are not willing to provide FP services to women transitioning from LAM to other FP methods until they go to the health facilities during their menses.”

Sociocultural and religious factors played an important role in influencing women’s MIYCN and FP practices and use of services. One woman noted, “I had planned to go to the health facility for tubal ligation after the birth of my sixth child; however, I had a dream where God questioned me about my plans. He told me that He was the giver and taker of life and that I should continue bearing children.” Partner and family support was also cited as critical for influencing adoption of recommended practices. Women expressed feeling a lack of support from their spouses and family members for EBF and FP use. One woman explained, “My grandmother discouraged us from exclusively breastfeeding our second born child. When the baby cried, we were told the baby was hungry, and he could not be sustained on breastmilk alone…After some time, my grandmother overpowered us and, when the child was 4 months, we introduced cow milk and some porridge.”

### Implications for Program Planning

Factors influencing success of integrated service delivery are outlined in Table 4. Based on learning generated from this experience, it is recommended that future replication efforts: build the leadership skills of sub-county teams to coordinate and oversee scale-up; assess facility readiness including availability of essential commodities; streamline data collection so that key indicators are included in MOH registers or rely on external assessments, such as client exit interviews and observations of provider–client interactions; build support among facility supervisors, health workers, and community leaders before implementation; ensure integrated training and mentoring in FP and MIYCN counseling and service delivery; and prioritize health centers and dispensaries, rather than hospitals, which provide a better platform for a “one stop shop” approach. For future efforts at higher-level health facilities, there is need to discuss strategies to address provider rotation and continuity of services with sub-county health management teams. There is also need for further strengthening provider capacity to counsel on and provide PPFP including long-acting and permanent methods and to counsel on the importance of continued breastfeeding for at least 2 years.

**Table 4:** Factors influencing the success of integrated service delivery

FACTORS that contributed to the success of integrated service delivery	FACTORS that hindered the success of integrated service delivery
• Advocacy and buy-in from national-level nutrition and reproductive health stakeholders • Involvement of the SCHMT and hospital management team at all levels of intervention design, planning, coordination, and implementation • Strategically designed behavior change communication materials and working tools, which made it easier for service providers and CHVs to convey standardized, accurate information to women and their families • Community and facility worker supervisor buy-in and leadership, oversight, and on-the-job mentoring • Involvement of community-level leaders, school administration, religious leaders, and other stakeholders as advocates for FP and nutrition • Mentorship and continuous engagement of health workers in provision of MIYCN-FP services, which led to retention of knowledge and skills • Human resource availability and continuity • Availability of FP methods/commodities and equipment • Whole site trainings, especially at facilities where staff rotations take place routinely	• Competing tasks and activities—for example, polio campaigns at the sub-county level delayed implementation of MIYCN-FP activities because priority was given to nationally mandated activities • Lack of team cohesion and transfer of learning at hospitals • Large training class sizes for CHVs, which may compromise the quality of learning and skills assessment • Large client load with staff shortages (especially at Bondo Sub-County Hospital, where provider concerns about client wait times often led them to shorten or skip the integration altogether) • Staff rotations, particularly at Bondo Sub- County Hospital, which resulted in trained staff being shifted to other units and a need for additional orientations for new staff • Inadequate male involvement from the outset of the program (male opposition was cited as key barrier to optimal MIYCN and FP practices) and lack of support from other family members including grandmothers and mothers-in-law • Cultural and religious factors • Infrastructural constraints—commodities, room privacy • Burdensome supplementary data collection mechanisms and gaps in data management among CHVs and facility providers

In spite of LAM’s effectiveness when practiced correctly, its reported practice in most countries (including Kenya) is extremely low (Moore et al. 2015). LAM relies on women understanding and following the criteria, however women who report using LAM often do not meet the criteria, remember the criteria, and/or transition to another modern method in a timely manner (Cooper et al. 2014; Fabic and Choi 2013; Kouyate et al. 2015; Sipsma et al. 2013). Further exploration of effective approaches for promoting LAM as a contraceptive option are needed.

At the community level, program implementers should consider engaging champions to promote optimal practices alongside CHVs, and incorporate specific activities to reach men, grandmothers, and other family and community members, who play an important role in influencing MIYCN and FP practices in Kenya (Nyanga et al. 2012). Approaches should be adapted as needed based on sociocultural factors unique to specific sites. Additionally, feedback should be solicited from mothers, fathers, and other community members on service provision in order to facilitate the development of client-centered approaches that promote an enabling environment for optimal practices. Experiences like the Bondo demonstration, where MIYCN and FP were integrated across the MNCH continuum of care, contribute to learning on approaches for enhancing delivery of holistic health services for women and children and reducing missed opportunities for care.

## Conclusion

This process learning makes an important contribution to the global dialogue on *how* to operationalize integration of services to reduce missed opportunities for care. It highlights that there are complexities that are not always fully comprehended at the start of the endeavor. The integration initiative demonstrates feasibility of integrating MIYCN and FP services at health centers and dispensaries, complemented by community-level integration activities. Additional small-scale replications with robust evaluation components are needed to assess impact on FP unmet need, exclusive breastfeeding, and other infant and young child nutrition outcomes and should be conducted in other parts of Kenya or other countries in the region with different cultural or social contexts before going to full scale. Changes in practices and use of services should be closely documented. Attention will be needed to monitor the adaptation that will inevitably be needed to fit with those new contexts.

## Footnotes

1 To use LAM, women must follow three criteria: exclusive breastfeeding; menstruation has not returned; and infant <6 months.

2 The most recent DHS available at the start of the intervention.

3 Catchment numbers drawn from program data.
